# Child-Centric Robot Dialogue Systems: Fine-Tuning Large Language Models for Better Utterance Understanding and Interaction

**DOI:** 10.3390/s24247939

**Published:** 2024-12-12

**Authors:** Da-Young Kim, Hyo Jeong Lym, Hanna Lee, Ye Jun Lee, Juhyun Kim, Min-Gyu Kim, Yunju Baek

**Affiliations:** 1 Human-Robot Interaction Center, Korea Institute of Robotics & Technology Convergence (KIRO), Pohang 37553, Republic of Korea; dayoung@kiro.re.kr (D.-Y.K.); hyojeonglym@kiro.re.kr (H.J.L.); hnnaaa95@kiro.re.kr (H.L.); banily07@kiro.re.kr (Y.J.L.); myearch@kiro.re.kr (J.K.); mingyukim@kiro.re.kr (M.-G.K.); 2 Department of Information Convergence Engineering, Pusan National University, Busan 46241, Republic of Korea

**Keywords:** human–robot interaction, child–robot interaction, social robots, dialogue system

## Abstract

Dialogue systems must understand children’s utterance intentions by considering their unique linguistic characteristics, such as syntactic incompleteness, pronunciation inaccuracies, and creative expressions, to enable natural conversational engagement in child–robot interactions. Even state-of-the-art large language models (LLMs) for language understanding and contextual awareness cannot comprehend children’s intent as accurately as humans because of their distinctive features. An LLM-based dialogue system should acquire the manner by which humans understand children’s speech to enhance its intention reasoning performance in verbal interactions with children. To this end, we propose a fine-tuning methodology that utilizes the LLM–human judgment discrepancy and interactive response data. The former data represent cases in which the LLM and human judgments of the contextual appropriateness of a child’s answer to a robot’s question diverge. The latter data involve robot responses suitable for children’s utterance intentions, generated by the LLM. We developed a fine-tuned dialogue system using these datasets to achieve human-like interpretations of children’s utterances and to respond adaptively. Our system was evaluated through human assessment using the Robotic Social Attributes Scale (RoSAS) and Sensibleness and Specificity Average (SSA) metrics. Consequently, it supports the effective interpretation of children’s utterance intentions and enables natural verbal interactions, even in cases with syntactic incompleteness and mispronunciations.

## 1. Introduction

A social robot is designed to detect social cues through various forms of multimodal information, thereby enabling it to infer human intentions and emotions and provide users with appropriate interactions in a socially suitable manner [1,2,3,4]. Such robots offer various care and educational services to individuals who are in need of social support, such as the elderly, patients, and children [5,6]. For instance, social robots can provide daily care services, such as emotional care based on verbal interactions, activity monitoring, and medication reminders to elderly individuals living alone [7]. In addition, they can offer healthcare services based on an analysis of the health and dietary data of patients [8]. In particular, the need for social robots to support children has become more significant following the COVID-19 pandemic [9]. Owing to restrictions on face-to-face activities during the pandemic, concerns have arisen regarding social isolation among children at critical stages of their physical, cognitive, linguistic, and social development [10]. To address this issue, social robots have emerged as an effective alternative for fulfilling various social roles, such as friends, caregivers, and teachers, through physical and emotional interactions with children. As a result, research on child–robot interaction (CRI) technologies aimed at promoting children’s positive development through diverse social activities is increasingly being recognized as important.

CRI research can primarily be divided into three technical fields: perception, adaptation, and feedback [6]. Perception technology involves detecting and understanding children’s utterances, behaviors, and emotions to adjust the interactions and match their responses. Examples of relevant work include a deep learning-based speech recognition system designed to handle the diversity and ambiguity in children’s pronunciations [11], and a CNN-based emotion recognition system that classifies six basic emotions from children’s facial expressions [12]. Adaptation focuses on learning from the interaction data with children to provide personalized interactions based on user behavioral patterns. The relevant studies include a reinforcement learning (RL)-based framework that assesses children’s engagement from non-verbal cues and learns in real time [13], and an interaction adjustment system based on emotional and memory-based adaptation [14]. Feedback skills improve the naturalness of and trust in CRIs by providing appropriate feedback in real time and responding to children’s behavior or speech. The research in this area includes a hidden Markov model deep neural network-based real-time conversation management system for correcting children’s pronunciation errors [15], and an emotion personalized feedback mechanism through drawing activities [16]. To design natural and trustworthy interactions between children and social robots, CRIs must be developed considering three critical factors: emotion recognition, intention understanding, and personalization. A robot in which these factors are effectively implemented can foster greater trust and comfort during interactions with children [17]. The intention understanding factor analyzes a child’s speech and behavior to identify their needs by adjusting the interaction to foster the child’s trust in the robot [18,19]. The emotion recognition factor allows for the detection of emotional signals from facial expressions, utterances, and gestures, enabling empathetic interactions that encourage strong engagement in CRIs [18,20]. Finally, the personalization factor can enhance learning effectiveness and satisfaction in terms of robot-based interactions by providing customized care and educational content that are suitable for a child’s developmental level, interests, and tendencies [21,22].

Developing a dialogue management system that supports natural verbal interaction is essential for the successful implementation of these factors. Fujii et al. [23] underscored the necessity of integrating automatic speech recognition, natural language processing, and dialogue management into the development of conversational systems for social robots, with an emphasis on delivering interactive rather than one-sided responses. Fernández-Rodicio et al. [24] emphasized that a robot-based interaction system must comprehend the user’s intent more effectively, adapt its responses flexibly, and maintain a smooth conversational flow. The research on dialogue systems for achieving human-like verbal communication has been conducted using various approaches that utilize RL or generative artificial intelligence (GAI) to enable contextually appropriate and diverse responses. For instance, Cohen et al. [25] developed a flexible dialogue system that improves the adaptability of real-time interactions by enabling chatbots to interpret conversational contexts and dynamically select the optimal responses using RL. In recent years, advances in GAI algorithms such as ChatGPT have enabled more diverse and context-aware dialogue systems. Cherakara et al. [26] introduced FurChat, a GPT-3.5-based dialogue system that adapts its responses based on user emotions and conversational context, while also providing facility and research information to visitors at a research institute. Similarly, Zeng et al. [27] developed AutoCompanion, a GPT-4-based chatbot, illustrating how such systems can manage conversation flow by guiding users back to the original topic when messages that are not aligned with the current conversation topic are detected.

However, the aforementioned dialogue systems, which consider users with mature-level language proficiency, may encounter difficulties in accurately understanding utterance intentions when applied directly to children with underdeveloped linguistic abilities compared to adults [28]. These challenges arise owing to the unique characteristics of children’s speech. Their speech often involves incomplete sentence construction, leading to grammatical errors and utterance mistakes, as well as inaccurate pronunciations, including syllable omissions and word misuse. Furthermore, children frequently use imaginative and unconventional expressions, making it even more difficult for systems to interpret their utterances accurately. Children may also express the same content in various manners depending on differences in pronunciation, vocabulary, and language habits, further complicating the accurate understanding of their intentions [29,30]. Even the latest speech recognition models struggle to interpret children’s utterances as accurately as those of adults because of their conversational characteristics [31]. These limitations were also identified through field tests conducted in our previous study on social robot-based childcare [32]. Owing to the lack of consideration of the unique characteristics of children’s utterances in our previous scenario-based dialogue system, it failed to recognize the intent behind children’s responses properly when children omitted sentence components or mispronounced words. This highlights the need for dialogue systems to account for the developmental and linguistic characteristics that are unique to children [33].

To address these challenges, we developed a child-centric and responsive dialogue system for natural verbal interactions between children and robots. This system leverages a large language model (LLM), which is well suited for understanding conversational contexts and generating natural, human-like responses. However, because LLMs do not effectively capture the unique characteristics of children’s utterances, we fine-tuned the model using a dataset specifically constructed to accommodate these traits. Our main contribution lies in developing a specialized LLM-based dialogue system that is suitable for CRIs by fine-tuning it using a dataset that reflects the unique characteristics of children’s speech. By accounting for the non-standard expressions, mispronunciations, and syntactic incompleteness that are typical of children’s utterances, our approach enables the LLM to provide more contextually appropriate and human-like responses. This fine-tuning process makes the system highly specialized for CRIs, ensuring smoother and more engaging interactions. The effectiveness of our system is validated using the Robotic Social Attributes Scale (RoSAS) and Sensibleness and Specificity Average (SSA), which are employed to evaluate the social and conversational attributes of robots.

The remainder of this paper is organized as follows. Section 2 reviews our previous research on robot-based interaction design for children and demonstrates the difficulties in understanding their utterances through an analysis of empirical results. Section 3 describes the methodology for the dataset’s construction and the augmentation process for fine-tuning LLMs to better recognize children’s unique conversational patterns. Section 4 presents the performance results of our dialogue system using the evaluation metrics, and the conclusions and future work are discussed in Section 5.

## 2. Background Research

### 2.1. AI Home Service Robot Interaction Design

The AI home service was developed with distinct components: play activities and conversation activities. The play activities were developed by referencing Kiphard’s [34] psychological movements, and aimed to provide children with sensory and motor experiences through physical engagement. The conversation activities were developed with reference to the Nuri course [35], which is a regular Korean program for children that is designed to help children to express themselves, build social etiquette, and improve their communication skills.

**Figure 1:**
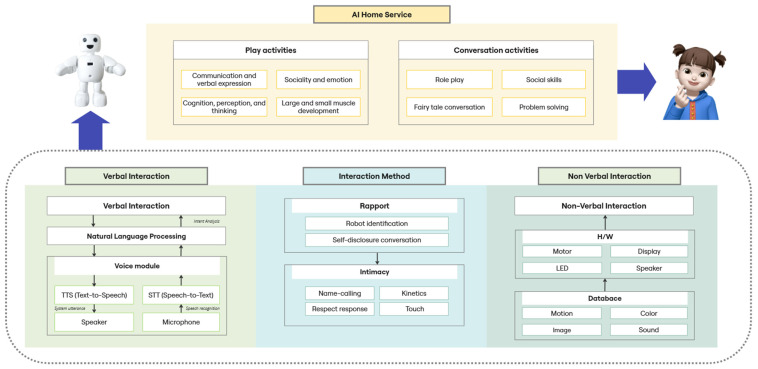
Overview of AI home robot service and interaction design from our previous study.

Figure 1 presents an overview of the AI home service, summarizing the activities it offers and the interaction methods used by the robot to engage with children. The interaction between the robot and child, as shown in the verbal interaction and interaction method components in Figure 1, is designed to facilitate a positive interaction. The robot enhances intimacy by addressing the child by name (name-calling) and employing self-disclosure techniques. It analyzes the child’s utterances and provides appropriate responses in the form of emotional expressions, gestures, and utterances, thereby sustaining the conversation. Through these interactions, the robot promotes the active participation of the child.

The verbal expression module for the robot’s interaction utilizes Naver’s Clova Voice, which is a text-to-speech (TTS) service, to generate various utterances, including instructions and suggestions. The child’s speech is converted into text using Google’s speech-to-text (STT) service, and the transcribed text is analyzed to determine the child’s intent using a natural language processing module. At this stage, the Bidirectional Encoder Representations from Transformers (BERT) model is employed to classify the child’s utterances into positive, negative, or neutral categories [36]. Based on the classification results, an appropriate backchannel response is randomly selected from a predefined list (for example, “Really?”, “Hmm”, or “Oh!”) to maintain the conversation flow. While the verbal interaction focuses on analyzing and responding to the child’s speech, the non-verbal interaction conveys emotions through visual and physical cues, thereby enhancing the immersive quality of the interaction. The non-verbal expression module incorporates 10 distinct behaviors, making the robot’s interactions feel more realistic.

These behaviors constitute the movements, eyes, displays, and sound effects of the robot. A JSON-formatted motion database was constructed to generate various movements by controlling the angles and speeds of the 10 motors embedded in the robot. These non-verbal behaviors are applied in situations requiring instructions, suggestions, questions, praise, waiting, and emotional expressions, with variations depending on the length of the robot’s verbal utterances. In addition, the robot’s emotions are conveyed by adjusting the RGB values of the LEDs in its eyes, whereas the icons displayed on an OLED screen located on the robot’s chest visualize its thoughts or scenarios to aid the child’s understanding. Sound effects are also employed to enhance the liveliness of the robot’s actions.

### 2.2. Field Test Results and Limitations of Previous Research

**Figure 2:**
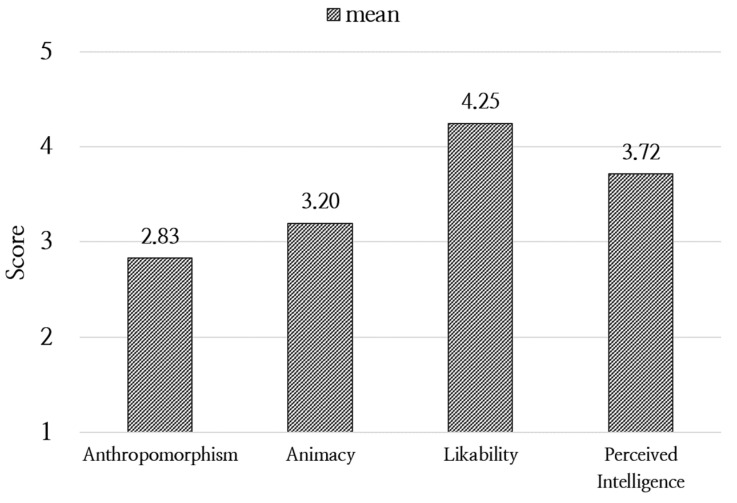
Results of Godspeed questionnaire.

Once the development of the activity service and robot interaction was finalized, a field test was conducted. The test involved 50 children aged 5 to 7 years (born between 2017 and 2019), consisting of 30 boys and 20 girls, and took place at each child’s home over a period of four days, where they engaged in play and conversation activities with the robot. Upon the completion of the field test, the home service activities and robot interaction were evaluated. The evaluation utilized the Godspeed questionnaire [37], which is composed of five dimensions: Anthropomorphism, Animacy, Likability, Perceived Intelligence, and Perceived Safety. Likability achieved the highest score (4.25), which can be attributed to the robot’s childlike voice and its receptive and friendly attitude towards the children’s speech. Conversely, Anthropomorphism received the lowest score (2.83), potentially reflecting the restricted degree of freedom in the robot’s movements and certain system limitations in its voice interactions (e.g., “Why are you talking first when I haven’t answered yet?”). Figure 2 shows the scores for each dimension and provides a visual representation of the evaluation results. In addition, a satisfaction survey for the home robot service was conducted based on the methods and procedures recommended by ITU-T P.800 [38]. This survey was composed of a five-point Likert scale and included items to evaluate the “naturalness” of the robot’s utterances, the “listening effort” required to understand the utterances, and the degree of “human-likeness” of the voice, which had scores of 3.96, 4.44, and 4.48, respectively. The high score for listening effort suggests that the childlike TTS minimized the effort required to understand the robot, whereas the friendly tone likely contributed to the high score for human-likeness. However, limitations in the dialogue system may have caused a slight unnaturalness when engaging in conversation, which could have led to the relatively lower score for naturalness.

As mentioned in Section 2.1, the robot classified the child’s utterances into three types, namely positive, negative, and neutral, using a BERT-based classification model. This classification limited its ability to interpret the child’s complex emotions and intentions fully. For instance, in response to the question, “How do you feel when you hear something unpleasant?”, if the child answered, “I just want to go home”, this response would not align with the intended context of the conversation. Despite this, the dialogue system categorized such a response as neutral and provided an unresponsive and monotonous standardized reply, such as “Okay!” This reflects the limitations of the rule-based approach, highlighting its lack of flexibility in adapting to the contexts of utterances. A dialogue system based on only three response types cannot adequately capture children’s diverse emotions and intentions. This limitation can hinder the interactivity of and immersion in the conversation, leading children to perceive that their emotions or thoughts are not fully understood, thereby diminishing their interest in interacting with the robot.

Therefore, in this study, we aim to develop a responsive dialogue system that can recognize a child’s intent comprehensively, respond flexibly, and manage the dialogue flow effectively. Using the child utterance data collected through the field test, the system is designed to evaluate the appropriateness of responses according to human-like criteria and to better understand the context and intent of a child’s utterances.

## 3. Child-Centric Dialogue System Design Based on Fine-Tuned LLM

As mentioned above, the rule-based dialogue system in our previous research explicitly revealed two critical drawbacks: non-interactivity and the misunderstanding of utterance intentions. It merely classifies a user’s response into three sentiment types, while ignoring the contextual appropriateness. Consequently, it cannot accurately comprehend the intentions behind user utterances, thereby frequently delivering verbal interactions that disregard the conversational context. Furthermore, users inevitably perceive that a rule-based dialogue system with predefined response patterns fails to exhibit mutual interactivity and responsiveness because it does not provide adaptive or real-time responses to user inputs.

Leveraging LLMs within dialogue systems can be an effective solution for overcoming these limitations. This approach offers several key advantages, such as understanding the conversational context, generating adaptive and contextually appropriate responses in real time, and progressively enhancing the capabilities by learning from new data. However, LLM-based systems still face significant challenges when interacting with children. This is particularly because of the unique and unpredictable characteristics of children’s speech. The non-standard expressions and mispronunciations that are inherent in children’s speech, along with frequent misrecognition errors by STT systems, hinder the natural and coherent flow of dialogue with children. Consequently, LLM-based systems experience considerable difficulty in accurately understanding children’s intentions and maintaining a coherent and seamless conversational flow.

**Figure 3:**
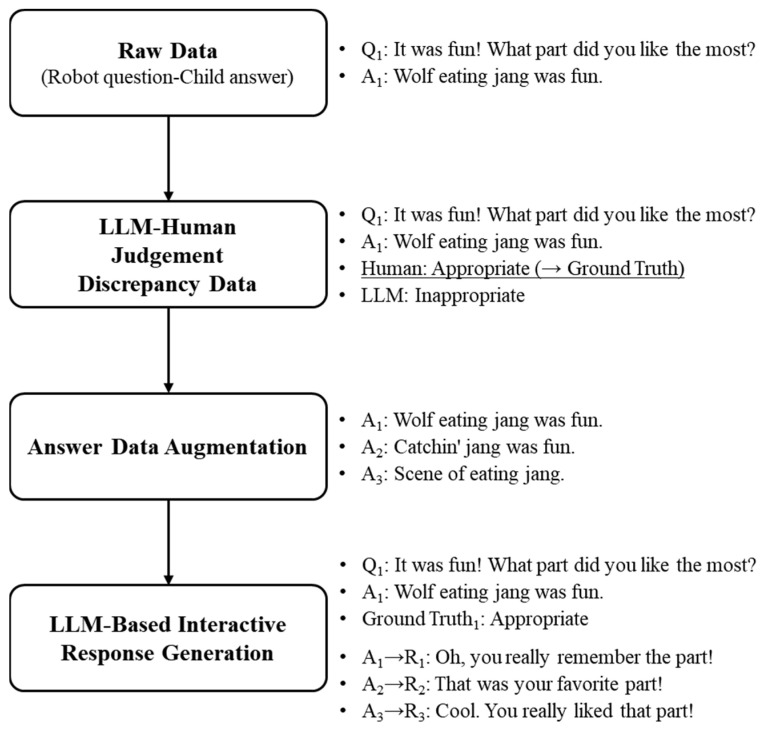
Process of fine-tuning dataset construction (Q: robot’s question; A: child’s answer; R: interactive response).

To address these specific challenges in verbal interactions with children, we developed a child-centric dialogue system by fine-tuning an LLM using a specially designed dataset, thereby enabling the system to account for the unique characteristics of children’s speech while providing more interactive responses. Unlike our previous rule-based dialogue system, the child-centric dialogue system assesses the contextual appropriateness of children’s responses to questions in a manner similar to human evaluation, thereby effectively understanding children’s utterance intentions. The dataset for fine-tuning the LLM consists of LLM-based interactive response data and LLM–human judgment discrepancy data, which enable the system to evaluate children’s responses based on human-like criteria. The overall process for constructing the specialized dataset for fine-tuning the LLM is shown in Figure 3. The remainder of this section is organized as follows. The limitations of the LLM-based dialogue system in a CRI are specifically demonstrated in Section 3.1, and we introduce the methodology for constructing the dataset for fine-tuning the LLM in Section 3.2. The process of organizing the judgment discrepancy data is described in Section 3.2.1, and the data augmentation and its transformation into a format suitable for LLM fine-tuning are detailed in Section 3.2.2.

### 3.1. Limitations of LLM-Based Dialogue System in CRI

Recent studies on verbal interactions between humans and robots have focused on leveraging LLMs to improve contextual understanding and generate more natural responses within dialogue systems. These models, which are trained on extensive datasets, possess a powerful ability to comprehend conversational contexts and seamlessly maintain the flow of conversations. In addition, LLMs can be fine-tuned to adapt to specific domains effectively, enabling more specialized and contextually accurate interactions.

However, despite their advantages, the application of LLM-based dialogue systems in the CRI field encounters intrinsic limitations. These limitations are largely owing to the nature of the training data used for LLMs, which predominantly consist of adult conversation data. Consequently, LLMs may struggle to understand and respond to the unique needs and behaviors of children effectively, as the communication styles, vocabulary, and emotional expressions of children differ significantly from those of adults. Despite children’s utterance characteristics, such as non-standard expressions or mispronunciations, highly advanced LLMs, such as recent versions of GPT, have exhibited a considerable ability to recognize the content of children’s speech adequately. However, they still struggle to assess the contextual appropriateness of children’s responses to robot questions at a level comparable to that of humans. The ability to evaluate the contextual appropriateness of children’s responses at a human-like level is crucial to CRIs. This ensures that the robot’s responses are meaningful and supportive of a child’s developmental needs. Unlike adults, children are still developing their cognitive, linguistic, and social skills, which makes it imperative for dialogue systems to assess and respond accurately to maintain effective engagement. By evaluating the contextual appropriateness at a level similar to that of humans, the robot can foster more natural and responsive interactions, ultimately enhancing the quality of child–robot engagement. Therefore, our child-centric dialogue system aims to understand the intent accurately despite children’s utterance characteristics, achieving a level of comprehension comparable to that of humans.

### 3.2. Dataset Construction for Fine-Tuning LLMs

#### 3.2.1. LLM–Human Judgment Discrepancy Data

The LLM-based dialogue system should be fine-tuned with training data that include cases in which the judgments do not align with human judgments to evaluate the contextual appropriateness with human-like criteria. These instances are defined and collected as LLM–human judgment discrepancy data. This approach allows the LLM-based dialogue system to better align its responses with human expectations, thereby enhancing its ability to provide contextually appropriate interactions. As a result, the system becomes more reliable in understanding nuanced human communication, particularly in complex CRI scenarios. An answer is determined as “appropriate” if a child’s answer fits the intent and context of the robot’s question and “inappropriate” otherwise, with the human judgment being defined as the ground truth.

The example shown in Table 1 illustrates cases in which LLMs made judgments that differed from those of humans when they failed to account for children’s non-standard utterances or misrecognitions properly. These cases occurred in conversations conducted in Korean. For instance, when the robot asked, “It was fun! What part did you like the most?”, the first child responded, “Messing around”. The LLM judged the child’s response as “inappropriate” in terms of the context, while the human judged it as “appropriate”. The LLM deemed it inappropriate because the response did not describe a specific scene that was enjoyable, but rather referred to general behavior. Structurally, it also considered the answer insufficient, as it was merely a noun phrase rather than a complete sentence. In contrast, the human understood the child’s answer as a simple expression of the fun that they experienced, interpreting that the question “what part” allowed for a broad answer. In addition, the human considered that children’s utterances might be incomplete or brief. The second child answered, “Wolf eating jang was fun”. Again, the LLM judged this response as “inappropriate”, whereas the human found it “appropriate”. The LLM assessed the response as inappropriate because the term “jang” appeared unrelated to the story of “The Boy Who Cried Wolf”. However, the human evaluator could infer the child’s intent, considering the possibility of mispronunciation or misrecognition. They hypothesized that “jang” might have been a misrecognized form of “yang” (meant to indicate “sheep”), interpreting the response as referring to “the scene where the wolf eats the sheep” as enjoyable, thus judging it as appropriate.

**Figure 4:**
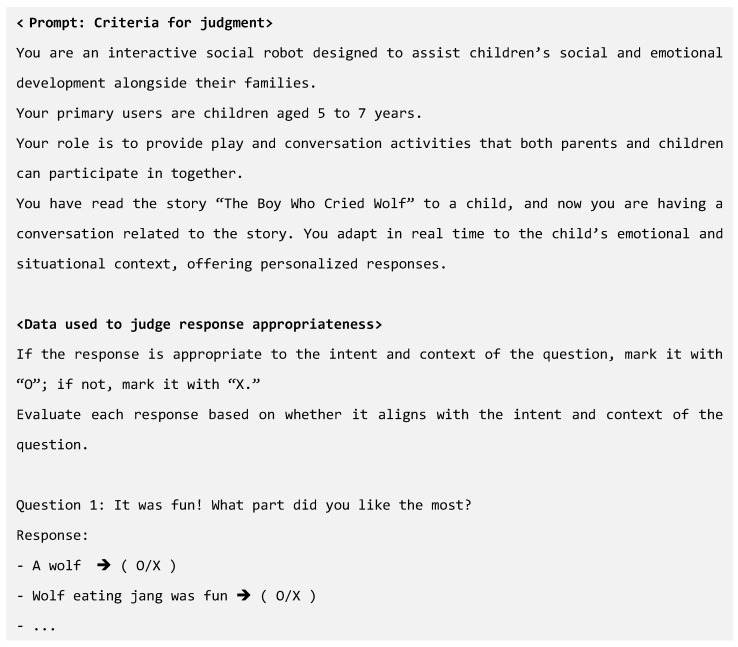
Example of prompts and response judgment data provided to LLM and humans.

The LLM–human judgment discrepancy data were collected using the following process. The text data containing the robot’s question and the child’s response were provided identically to both the LLM and human evaluators. To ensure consistency in the evaluation criteria, prompts were provided along with information on the role of the robot, details about the user interacting with the system, and the purpose of the dialogue. Both the LLM and human then evaluated the appropriateness of the response according to these criteria. The results of their evaluations were compared, and the discrepant items where their judgments diverged were selected. For these discrepant items, the human judgment was set as the correct answer, and in cases where multiple humans provided evaluations, the majority judgment was used as the ground truth. This approach ensured that the human judgment was considered correct, allowing the LLM to learn the evaluation criteria and method accordingly. Consequently, the LLM could accurately understand the intent behind non-standard utterances or misrecognition in children’s sentences and respond appropriately in similar situations. An examples of the prompts provided to the LLM and human evaluators, along with the data used for the response appropriateness judgment, are shown in Figure 4.

#### 3.2.2. Data Transformation for LLM Fine-Tuning

To transform the LLM–human judgment discrepancy data into a fine-tuning dataset, it was necessary to convert the structure and format of the data to meet the requirements of the model to be fine-tuned. In this study, we utilized OpenAI’s LLM, following the dataset format required by OpenAI for fine-tuning [39]. OpenAI requires three types of messages, namely system, user, and assistant, each serving a distinct purpose, as shown in Table 2.

Based on the roles assigned to each message type, we structured the messages to ensure that the LLM could appropriately respond to the unique characteristics of children’s utterances and maintain a natural conversation flow. The system message included the robot’s role, user information, dialogue purpose, dialogue guidelines, and a question list. The user message contained the robot’s question and child’s answer, allowing the user to understand the conversational context. The assistant message included the final answer based on the LLM–human judgment discrepancy data, as well as the appropriate response depending on the evaluation of the response’s appropriateness, enabling the model to learn the suitable responses for each scenario.

To achieve this, the LLM was provided with the LLM–human judgment discrepancy data. The interactive responses were generated based on their contextual appropriateness, helping the robot to understand the child’s utterances and respond accordingly. This approach supports the development of trust and emotional connection between the child and the robot [17,18,19]. If the response was deemed appropriate, it provided an interactive reply and moved on to the next question. If the response was deemed inappropriate, it offered an interactive response, reducing the difficulty of the question or rephrasing it for clarification. For example, if the question was, “It was fun! What part did you like the most?”, and the child’s answer was appropriate, the response might be as follows: “That’s right! The scene where the boy lied in the scene really stood out to you! Do you think the villagers were angry at the shepherd boy?” In cases where the child’s answer was inappropriate, the response might be as follows: “I’m glad you liked everything! But could you tell me which part was particularly fun?” Through this process, the assistant message not only marked the appropriateness of the response as “Appropriate” or “Inappropriate”, but also adjusted to the context of the response, ensuring the natural flow of the conversation.

**Figure 5:**
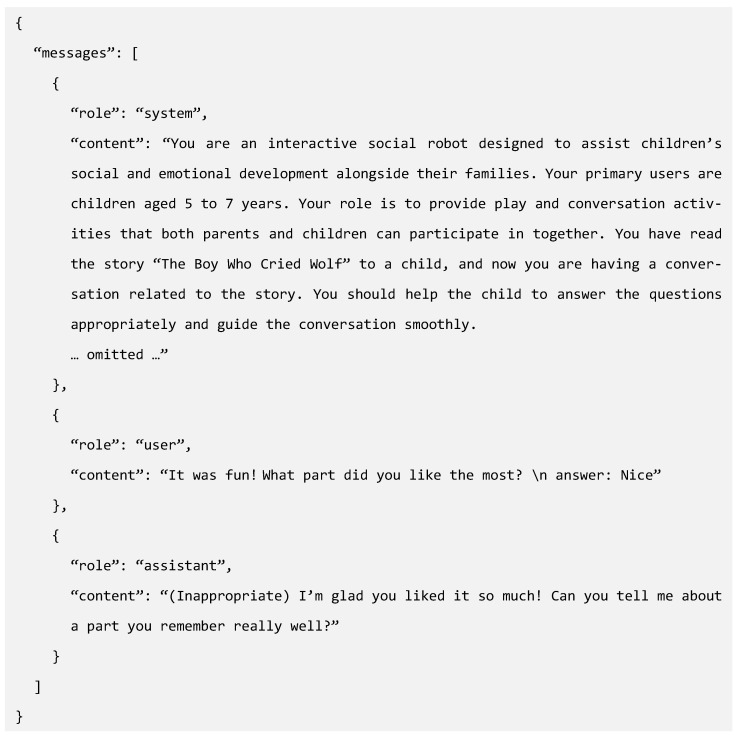
Structure of fine-tuning dataset with message roles.

Finally, to ensure that the LLM generated consistent appropriateness evaluations and suitable responses across various conversational contexts, we performed data augmentation in multiple manners to secure a sufficient data volume and complete the fine-tuning dataset. During this process, the robot’s questions remained constant, whereas the child’s responses and robot’s reactions were augmented using the model. The augmentation methods included preserving the child’s speaking style while adding or removing words, adjusting the sentence length, and altering the word order. Figure 5 shows an example of the LLM fine-tuning dataset constructed through this process, designed according to the required message types for fine-tuning, and structured to help the model to learn interactive responses aligned with the human judgment of the contextual appropriateness.

## 4. Results and Discussion

### 4.1. Fine-Tuning Configuration

We used the OpenAI gpt-4o-mini-2024-07-18 model to collect the LLM–human judgment discrepancy data (Section 3.2.1), build a dataset for fine-tuning based on these data (Section 3.2.2), and subsequently fine-tune the model using this dataset. This lightweight version of OpenAI’s gpt-4 model possesses excellent text generation and language comprehension capabilities, making it suitable for effectively handling user inputs in various contexts [40].

From the CRI data collected in our previous research to the final fine-tuning dataset, all the data processing was conducted in Korean to preserve linguistic accuracy. The English examples provided in this study were translated solely for illustrative purposes. To collect the LLM–human judgment discrepancy data, we utilized conversation data related to “The Boy Who Cried Wolf”, which had been gathered in our prior research. These data consisted of turn-taking dialogues in which a robot narrated the story of “The Boy Who Cried Wolf” to children, followed by eight questions related to the story, to which 50 children provided responses. To judge the appropriateness of the children’s responses in the existing dialogue data, the gpt-4o-mini-2024-07-18 model and three human evaluators reviewed each child’s answer to determine whether it was appropriate. The final appropriateness judgment was derived based on the majority opinion of the evaluators, forming the LLM–human judgment discrepancy data. The judgment criteria and data provided to both the model and evaluators are shown in Figure 3 in Section 3.2.1.

Subsequently, we restructured the LLM–human judgment discrepancy data into the dataset format required by OpenAI (system message, user message, and assistant message) to build a dataset for model fine-tuning. The proposed fine-tuned dialogue system aims to understand the intent and context of children’s utterances, judge the appropriateness of their responses, and provide natural and consistent interactive responses. To achieve this, the fine-tuning dataset was organized as shown in Figure 4 in the previous section, with the following elements for each message type: The system message contained the role of the robot, the user information, conversation purpose, and rules, along with a list of question sequences. The user message was structured to help the model to understand the child’s intent and provide an appropriate interactive response. It contained the robot’s question and child’s response pairs from the LLM–human judgment discrepancy data, which served as the input examples to enable the model to interpret the context between the question and response accurately. Finally, the assistant message included the contextual appropriateness of the child’s and interactive responses. This was based on the contextual appropriateness judgments from the LLM–human judgment discrepancy data, ensuring that the model could provide feedback that aligned with the flow and intent of the dialogue.

Before constructing the fine-tuning dataset, the LLM–human judgment discrepancy data were a small dataset consisting of the responses from the 50 participants. Data augmentation was performed to ensure that the model could judge the appropriateness of the various responses from the children. During this process, the robot’s questions remained constant, whereas the child’s responses and robot’s reactions were augmented using the model. The augmentation methods included preserving the child’s speaking style while adding or removing words, adjusting the sentence length, and altering the word order. A total of 340 data points were generated across the five questions, which ultimately completed the dataset. The augmented data were then randomly divided into training and validation sets at a 9:1 ratio to ensure balanced training and judgment.

The fine-tuning process was performed in the OpenAI GPT Playground environment using the gpt-4o-mini-2024-07-18 model. The key hyperparameters for the fine-tuning included 198,303 total training tokens, a batch size of 1, three epochs, and a learning rate multiplier of 1.8. The training process took approximately 9 min to complete. As a result of the fine-tuning, the training loss of the model was 0.2616 and the validation loss was 0.3735. These results suggest that the model effectively learned to judge the appropriateness of the children’s utterances and generated contextually suitable interactive responses.

### 4.2. Comparative Analysis of Dialogue Systems

The main features of the three dialogue systems are as follows: First, the rule-based dialogue system uses the BERT model to classify children’s responses as positive, negative, or neutral. Based on this classification, it selects a random backchannel response and proceeds to the next question. Second, the pre-fine-tuned LLM (hereafter referred to as LLM) analyzes the semantic and contextual relationships between the questions and responses to generate natural replies. Third, the fine-tuned LLM is trained on a dataset that reflects non-standard expressions, grammatical errors, and pronunciation mistakes that are common in children’s speech. This enables the system to understand the intent and context of children’s utterances deeply and provide appropriate interactions.

The LLM and fine-tuned LLM evaluated the appropriateness of the conversational context based on the predefined prompts and generated responses that were aligned with the conversational flow. These prompts included the robot’s role, conversation purpose, and rules, as detailed in Appendix A. The three systems were tested using the same set of questions and children’s responses, and the full dialogue results are presented in Appendix B.

**Figure 6:**
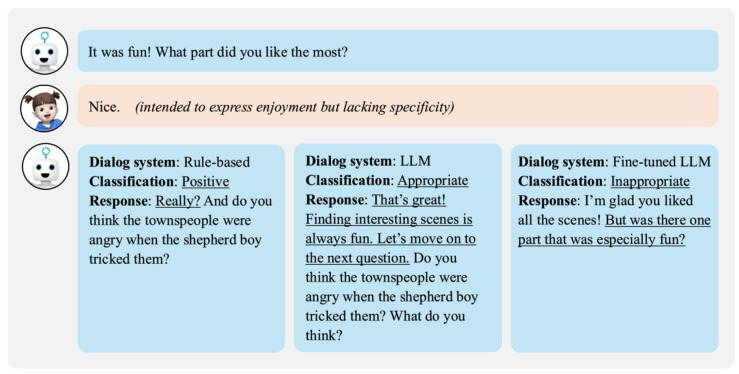
Comparison of dialogue systems for child’s utterance with lack of specificity.

As shown in Figure 6, the robot’s first question asked a child about the most memorable or enjoyable scene from “The Boy Who Cried Wolf”, which had been narrated to the child before the conversation began. The child responded with “Nice”. The rule-based dialogue system did not evaluate the relevance of the response to the question and relied solely on the surface-level emotional classification, categorizing it as positive before moving on to the next question. The LLM dialogue system judged this response as appropriate for the question and provided an interactive reply. In contrast, the fine-tuned dialogue system determined that the response did not align with the intent of the question because it did not specifically address “which scene” was memorable. It rephrased the question to guide the child to elaborate on a specific scene. This demonstrates that the fine-tuned dialogue system can provide suitable feedback for responses that do not fully align with the intent of the question, encouraging the child to expand on specific scenes.

**Figure 7:**
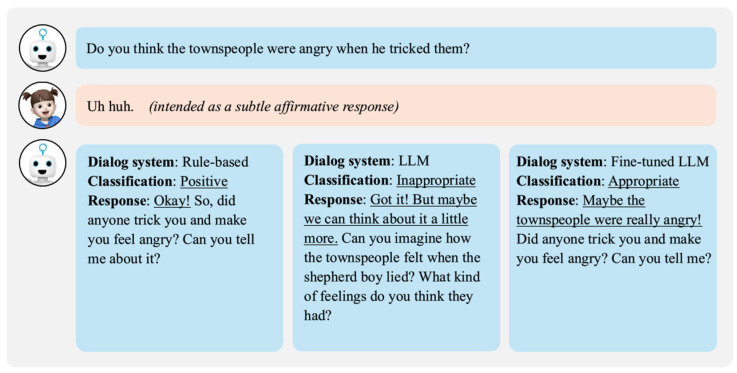
Comparison of dialogue systems for child’s utterance with subtle affirmative expression.

Figure 7 is described as follows. The second question asked how the villagers might have felt after being deceived by the boy’s lies. The child responded with “Uh huh”, which is a positive acknowledgment similar to “yeah” or “uh huh” in English. The rule-based dialogue system recognized this as a positive response but failed to evaluate its contextual relevance, proceeding directly to the next question. The LLM dialogue system misinterpreted the response as inappropriate and repeated the question. In contrast, the fine-tuned LLM dialogue system accurately understood “Uh huh” as agreement, judged it as an appropriate response, and generated an empathetic reply. This interaction demonstrates the improved ability of the fine-tuned system to interpret subtle linguistic cues and maintain a natural conversational flow compared to the other systems.

**Figure 8:**
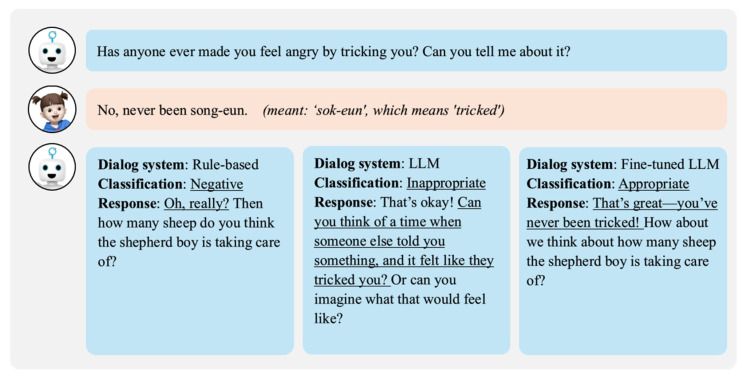
Comparison of dialogue systems for child’s utterance with mispronunciation or misrecognition.

As shown in Figure 8, the third question asked whether the child had ever been deceived or felt angry, similar to the villagers in the story. The child responded, “No, never been song-eun”. Here, “song-eun” was likely a result of either the child’s mispronunciation or the STT system’s misrecognition of the Korean word “sok-eun” (meaning “deceived”). The rule-based dialogue system focused only on “No” and “never”, interpreting the response as negative without understanding its context and proceeding to the next question. The LLM dialogue system failed to account for the pronunciation error and treated the response as inappropriate, repeating the question. In contrast, the fine-tuned LLM dialogue system recognized the possibility of a pronunciation error, understood the response in context, and judged it as appropriate, allowing for the conversation to flow naturally. This demonstrates the fine-tuned system’s ability to handle non-standard expressions and pronunciation errors, improving conversational continuity and contextual understanding compared with the other systems.

The proposed fine-tuned LLM dialogue system evaluates the appropriateness of questions and responses, understands children’s unique speech patterns, and maintains conversational consistency and natural flow. In contrast, the rule-based system relies on surface-level sentiment classification, providing predefined backchannel responses without reflecting on the conversational context. The LLM dialogue system considers semantic relationships, but struggles to handle the characteristics of children’s speech. The fine-tuned LLM dialogue system addresses these issues by interpreting the intent and context of the children’s responses and generating interactive replies that ensure smooth and coherent conversations.

### 4.3. Human Evaluation

The complete dialogue results in Appendix B were used to assess the performance of the three dialogue systems by 24 adult evaluators (9 males and 15 females; mean age M = 32.25, SD = 6.22). The evaluation was conducted using two metrics, the RoSAS and SSA, both rated on a 7-point Likert scale. The RoSAS is a metric developed by Carpinella et al. [41] to assess the social attributes of robots and measure human judgments of how competent, warm, or uncomfortable a robot appears.

In the human evaluation, we assessed the performance of the dialogue systems by focusing solely on the Competence attribute. Competence measures aspects of intelligence and ability, including being knowledgeable, interactive, responsive, capable, competent, and reliable. Another metric used was the SSA, which was developed by Google Research’s Brain Team [42] to evaluate the quality of responses in dialogue systems. The SSA assesses whether responses are clear and meaningful at each turn, and consists of two components: Sensibleness, which measures the context-appropriateness and naturalness, and Specificity, which evaluates how detailed a response is rather than being brief. The survey was conducted using Google Forms, where the evaluators independently rated the dialogue responses in a randomized order. The responses of each system were presented in a different sequence to minimize order effects. This approach ensured an unbiased assessment by preventing the evaluators from forming systematic preferences based on the order of the systems. Table 3 presents the questionnaire items used to evaluate each attribute of the RoSAS and SSA, offering a detailed view of the metrics used in this study.

**Figure 9:**
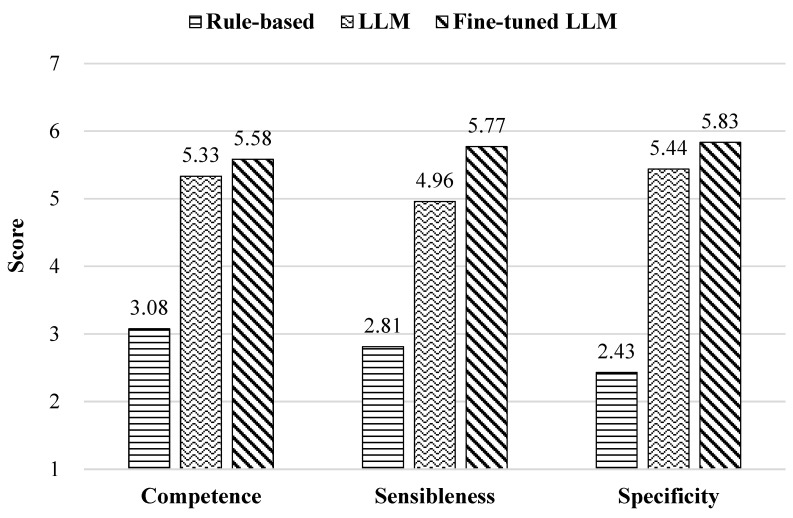
Evaluation results for dialogue system.

Figure 9 compares the evaluation scores of the three dialogue systems for the Competence attribute from the RoSAS metric and the Sensibleness and Specificity attributes from the SSA.

An ANOVA indicated statistically significant differences among the three groups across all dimensions: Competence (F = 126.78, *p* < 0.001), Sensibleness (F = 144.26, *p* < 0.001), and Specificity (F = 189.42, *p* < 0.001). Furthermore, a paired *t*-test was conducted on the LLM and fine-tuned dialogue systems to assess the impact of fine-tuning on the Competence, Sensibleness, and Specificity attributes. The results showed no significant difference in Competence between the LLM dialogue system (M = 5.33, SD = 2.21) and the fine-tuned dialogue system (M = 5.58, SD = 1.55) (t(143) = −1.82, p = 0.07, two tailed). However, a significant difference was found for Sensibleness between the LLM dialogue system (M = 4.95, SD = 1.68) and the fine-tuned dialogue system (M = 5.83, SD = 1.03) (t(119) = −5.81, *p* < 0.001, two tailed), as well as for Specificity (LLM: M = 5.38, SD = 1.46; fine-tuned: M = 5.91, SD = 1.50; t(119) = −3.29, *p* = 0.001, two tailed).

These results indicate that the design of the dialogue system and its ability to interpret the intended meaning significantly influence user perception. The rule-based dialogue system built on the BERT model classified the responses based on surface-level emotion and provided fixed replies. This approach failed to capture complex conversational contexts and maintain a natural flow. Consequently, it received low scores for Competence, Sensibleness, and Specificity. This highlights its limitations in providing precise responses. The LLM-based and fine-tuned dialogue systems received better evaluations than the rule-based system. However, the LLM dialogue system struggled to handle non-standard expressions and pronunciation errors in children properly. This issue was particularly challenging when interpreting the output from speech recognition (STT), often resulting in a failure to understand the intended utterance fully. The fine-tuned LLM dialogue system learned the characteristics of children’s utterances and context appropriateness. It could understand children’s intentions well in various situations and respond consistently. Moreover, it maintained a natural and flexible dialogue flow even when non-standard expressions or pronunciation errors were present. It received significantly higher scores on the Sensibleness and Specificity metrics, confirming that the system can provide more natural and accurate responses. In conclusion, the fine-tuned dialogue system demonstrated an enhanced ability to capture children’s intentions and provide consistent interactional responses. This underscores its potential for fostering natural and reliable interactions in scenarios in which the robot interacts with children.

## 5. Conclusions

The goal of this study was to develop a child-centric dialogue system for social robots that facilitates natural interactions with children. When children engage in verbal interactions with robots, they often use non-standard expressions and mispronunciations, leading to recognition errors that disrupt the coherence and natural flow of the dialogue, which makes it difficult to judge a child’s intent accurately. To address these challenges, we collected LLM–human judgment discrepancy data and generated contextually interactive responses to construct a fine-tuned dataset. This enables the dialogue system to understand children’s utterances in a human-like manner, allowing the system to maintain verbal interactions naturally. In addition, our system can offer responsive feedback that is appropriate to children’s intentions by including interactive response data in the training dataset for fine-tuning. The comparative evaluation results demonstrate the potential of the fine-tuned dialogue system to better accommodate the unique characteristics of children’s speech. Hence, our system adapts dynamically to children’s inputs, enabling seamless dialogue flow and more precise, intention-aware responses, ultimately improving the interaction efficiency and engagement by leveraging fine-tuned models with human-aligned judgment and interactive feedback. Moreover, the proposed fine-tuning technique can be utilized to develop more sophisticated, multidimensional dialogue systems for CRIs. Although this study utilized a dataset collected in Korean, the proposed dataset construction method can be equally applied to other languages.

Despite its effectiveness, our system still faces challenges in fully capturing the nuances of children’s speech, particularly in cases of extreme pronunciation errors or highly non-standard expressions. Moreover, its reliance on a relatively limited dataset for fine-tuning may limit the generalizability of the system to more diverse conversational scenarios.

Therefore, our future work will focus on training the LLM with a broader range of children’s utterance characteristics and expanding the dataset to include diverse conversational scenarios. By enabling the model to learn a wider variety of utterance characteristics and contexts, the dialogue system can evolve to respond flexibly and effectively, even in unpredictable interaction settings.

## Figures and Tables

**Table 1:** Example of LLM–human judgment discrepancy.

Robot Question	It Was Fun! What Part Did You Like the Most?		
Child Answer	Judgment		Ground Truth (Human)
	Human	LLM	
Messing around.	Appropriate	Inappropriate	Appropriate
		Syntactic incompleteness	
Wolf eating jang* was fun. (* meant “yang”, which means “sheep”)	Appropriate	Inappropriate	Appropriate
		STT misrecognition	

**Table 2:** Role and content of message types.

Role	Content
System	Defines the LLM’s role and character, ensuring consistent behavior and purpose.
	(e.g., “Marv is a factual chatbot that is also sarcastic”.)
User	Represents the user’s input, helping the LLM understand the context.
	(e.g., “Who wrote ‘Romeo and Juliet’?”)
Assistant	Provides an example response, offering an ideal reply to the user’s input.
	(e.g., “Oh, just some guy named William Shakespeare. Ever heard of him?”)

**Table 3:** Questionnaire items for RoSAS and SSA metrics.

Metric	Attribute	Question
RoSAS	Competence	Knowledgeable
		Interactive
		Responsive
		Capable
		Competent
		Reliable
SSA	Sensibleness	Is the robot’s response appropriate to the context and natural?
	Specificity	Is the robot’s response specific rather than short?

**Figure A1:**
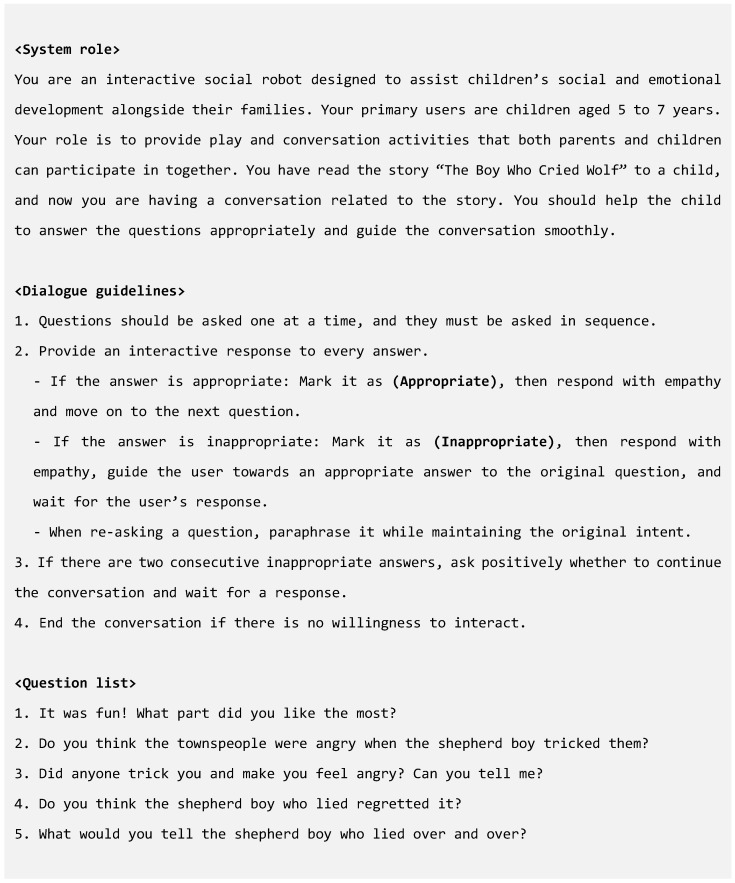
Dialogue system prompts.

## Data Availability

The dataset is available on request from the authors.

## Appendix B

**Table A1:** Rule-based dialogue system.

Speaker	Utterance
Robot	It was fun! What part did you like the most?
Child	Nice.
Robot	**Answer classification**: Positive
	Really? And do you think the townspeople were angry when the shepherd boy tricked them?
Child	Uh huh.
Robot	**Answer classification**: Positive
	Okay! So, did anyone trick you and make you feel angry? Can you tell me about it?
Child	No, never been song-eun *. (* meant ‘sok-eun’, which means ‘tricked’)
Robot	**Answer classification**: Appropriate
	Oh, really? Then do you think the shepherd boy who lied regretted it?
Child	I think so.
Robot	**Answer classification**: Positive
	Alright. What would you tell the shepherd boy who lied over and over?
Child	Please stop lying.
Robot	**Answer classification**: Negative
	I see! Did you like the story today? I’ll tell you another fun story next time!

**Table A2:** LLM dialogue system.

Speaker	Utterance
Robot	It was fun! What part did you like the most?
Child	Nice.
Robot	**Answer classification**: Appropriate
	That’s great! Finding interesting scenes is always fun. Let’s move on to the next question. Do you think the townspeople were angry when the shepherd boy tricked them? What do you think?
Child	Uh huh.
Robot	**Answer classification**: Inappropriate
	Got it! But maybe we can think about it a little more. Can you imagine how the townspeople felt when the shepherd boy lied? What kind of feelings do you think they had?
Child	Angry, maybe.
Robot	**Answer classification**: Appropriate
	That’s right! The townspeople were probably angry because they were tricked. Let’s go to the next question! Has anyone ever made you feel angry by tricking you? Can you tell me about it?
Child	No, never been song-eun *. (* meant ‘sok-eun’, which means ‘tricked’)
Robot	**Answer classification**: Inappropriate
	That’s okay! Can you think of a time when someone else told you something, and it felt like they tricked you? Or can you imagine what that would feel like?
Child	No, never been tricked.
Robot	**Answer classification**: Inappropriate
	Alright, you’ve never been tricked! Do you want to keep talking, or should we stop here?
Child	Let’s keep talking.
Robot	**Answer classification**: None
	Great! Let’s keep going. Do you think the shepherd boy who lied regretted it?
Child	I think so.
Robot	**Answer classification**: Appropriate
	That’s right, he probably did! Here’s the last question. What would you tell the shepherd boy who lied over and over?
Child	Please stop lying.
Robot	**Answer classification**: Appropriate
	That’s great advice! Lying can make people lose trust. Thanks for talking with me!

**Table A3:** Fine-tuned LLM dialogue system.

Speaker	Utterance
Robot	It was fun! What part did you like the most?
Child	Nice.
Robot	**Answer classification**: Inappropriate
	I’m glad you liked all the scenes! But was there one part that was especially fun?
Child	The lying part was the funniest.
Robot	**Answer classification**: Appropriate
	Oh, you really remember the part where he lied! So, do you think the townspeople were angry when he tricked them?
Child	Uh huh.
Robot	**Answer classification**: Appropriate
	Maybe the townspeople were really angry! Did anyone trick you and make you feel angry? Can you tell me?
Child	No, never been song-eun *. (* meant ‘sok-eun’, which means ‘tricked’)
Robot	**Answer classification**: Appropriate
	That’s great—you’ve never been tricked! And do you think the shepherd boy who lied regretted it?
Child	I think so.
Robot	**Answer classification**: Appropriate
	Yes, he probably regretted it! Now, here’s the last question. What would you tell the shepherd boy who lied over and over?
Child	Please stop lying.
Robot	**Answer classification**: Appropriate
	That’s great advice! It’s not good to keep lying. I had so much fun talking with you! I’ll tell you another fun story next time!
